# Aedesin: Structure and Antimicrobial Activity against Multidrug Resistant Bacterial Strains

**DOI:** 10.1371/journal.pone.0105441

**Published:** 2014-08-27

**Authors:** Sylvain Godreuil, Nadia Leban, André Padilla, Rodolphe Hamel, Natthanej Luplertlop, Aurélie Chauffour, Marion Vittecoq, François Hoh, Frédéric Thomas, Wladimir Sougakoff, Corinne Lionne, Hans Yssel, Dorothée Missé

**Affiliations:** 1 Centre Hospitalier Régional Universitaire de Montpellier, Hôpital Arnaud de Villeneuve, Département de Bactériologie-Virologie, Montpellier, France; 2 Centre d'études d'agents Pathogènes et Biotechnologies pour la Santé, CNRS-UMR 5236/UM1/UM2, Montpellier, France; 3 Centre de Biochimie Structurale Inserm U1054, CNRS UMR5048, Montpellier, France; 4 Laboratoire MIVEGEC, UMR 224 IRD/CNRS/UM1, Montpellier, France; 5 Department of Microbiology and Immunology, Faculty of Tropical Medicine, Mahidol University, Bangkok, Thailand; 6 Centre d'Immunologie et des Maladies Infectieuses, Inserm U1135, Sorbonne Universités, UPMC, APHP Hôpital Pitié-Salpêtrière, Paris, France; 7 Centre de Recherche de la Tour du Valat, le Sambuc, Arles, France; Academia Sinica, Taiwan

## Abstract

Multidrug resistance, which is acquired by both Gram-positive and Gram-negative bacteria, causes infections that are associated with significant morbidity and mortality in many clinical settings around the world. Because of the rapidly increasing incidence of pathogens that have become resistant to all or nearly all available antibiotics, there is a need for a new generation of antimicrobials with a broad therapeutic range for specific applications against infections. Aedesin is a cecropin-like anti-microbial peptide that was recently isolated from dengue virus-infected salivary glands of the *Aedes aegypti* mosquito. In the present study, we have refined the analysis of its structural characteristics and have determined its antimicrobial effects against a large panel of multidrug resistant bacterial strains, directly isolated from infected patients. Based the results from nuclear magnetic resonance spectroscopy analysis, Aedesin has a helix-bend-helix structure typical for a member of the family of α-helix anti-microbial peptides. Aedesin efficiently killed Gram-negative bacterial strains that display the most worrisome resistance mechanisms encountered in the clinic, including resistance to carbapenems, aminoglycosides, cephalosporins, 4^th^ generation fluoroquinolones, folate inhibitors and monobactams. In contrast, Gram-positive strains were insensitive to the lytic effects of the peptide. The anti-bacterial activity of Aedesin was found to be salt-resistant, indicating that it is active under physiological conditions encountered in body fluids characterized by ionic salt concentrations. In conclusion, because of its strong lytic activity against multidrug resistant Gram-negative bacterial strains displaying all types of clinically relevant resistance mechanisms known today, Aedesin might be an interesting candidate for the development of alternative treatment for infections caused by these types of bacteria.

## Introduction

Antibiotics have saved millions of lives worldwide by significantly decreasing the mortality associated with infectious diseases. However, these drugs are losing their effectiveness because of increasing antimicrobial resistance, as their massive and repetitive use in human and veterinary medicine has resulted in the emergence of multidrug-resistant (MDR) strains of bacteria that has become a serious global problem without any signs of abating. The propensity of microbes to develop multidrug-resistance is a natural trait following billions of years of evolution. Indeed, widespread resistance against several types of modern synthetic antibiotics has been discovered among bacterial strains that had been geologically isolated from the surface of the earth for more than 4 millions years [1], demonstrating that mechanisms of antibiotic modification and inactivation are part of the highly specific evolutionary adaptations of these microorganisms to evade the cytotoxic action of antibiotics, even those they have yet to encounter.

Particularly worrisome is the emergence of methicillin-resistant *Staphylococcus aureus* (MRSA) and *Enterococcus faecium*, glycopeptide-resistant Enterococcus (GRE), as well as MDR Gram-negative enterobacteria, in particular *Escherichia coli*, *Klebsiella pneumonia, Acinetobacter baumanii* and *Pseudomonas aeruginosa*, that, because of their production of broad-spectrum β-lactamases, i.e. AmpC cephalosporinase overproduction and extended spectrum β-lactamase, have become resistant to the third generation of cephalosporins [2]. The more recent emergence and expansion of the so-called carbapenemases determining resistance to carbapenems, a class of antibiotics of last resort for many bacterial infections, is also a cause of concern since these enzymes are presently found in the four known classes of β-lactamases (class A, B, C and D) and are determined by genes frequently harbored on highly transferable plasmids, in particular those coding for the carbapenemases KPC (class A), VIM and NDM (class B), and OXA-48 (class D) (see Table S1 for the corresponding resistance profiles). As resistance towards antibiotics becomes more common, there is an increased need for alternative treatments. However, novel antibiotics are not being developed at anywhere near the pace necessary to keep ahead of the natural ability of bacteria to evolve and defend themselves against antibiotics and, in addition, there has been a continued decline in the number of newly approved drugs [3]. Therefore, in addition to better management of antibiotic use, there is an urgent need for the development of novel therapeutic approaches to treat infections with MDR bacterial strains.

Ubiquitous in nature, antimicrobial peptides (AMP) are a unique and diverse group of molecules that were initially identified in insects and that form an important component of the innate immune system in all living organisms [4], [5]. AMP typically have broad spectrum activity against pathogenic bacteria and fungi, with various modes of action that may differ among bacterial species. Based on structure-function relationship, these peptides with a length between 12 and 50 amino acids can be divided in three classes based on their secondary structure: α-helical peptides, β sheet peptides - or mixed structures - and so-called extended peptides that do not fold into regular secondary structure elements and that often contain high proportions of certain amino acids, specifically Arg, Trp or Pro residues [6]. Most AMP carry a cationic charge that promotes selective interaction with negatively charged bacterial membranes, rather than zwitterionic mammalian cell surfaces. In addition, they contain amphipathic domains which facilitate their interaction with fatty acyl acids, thereby enabling them to associate with membranes, which is a definite property of these peptides. Many linear AMP are unstructured in aqueous solution and require a membranous environment to adopt such a stable, amphipathic, conformation. As most bacterial surfaces are anionic, the initial contact between the peptide and the target organism is electrostatic. Their amino acid composition, amphipathicity, cationic charge and size allow the AMP to attach to and insert into membrane bilayers to form pores by ‘barrel-stave’, ‘carpet’ or ‘toroidal-pore’ mechanisms [7]. In contrast to many conventional antibiotics, AMP appear to be bactericidal [8] instead of bacteriostatic, although in many cases, the exact mechanism of killing is not known [7]. Because of their particular mode of action, the antimicrobial properties of AMP have raised clinical attention and research interest over the past years [9]. Importantly, natural AMP have co-evolved with bacterial strains and their ability to permeabilize cytoplasmic membranes is less prone to the development of resistance, such as changes in the molecular charge of cell surface proteins or proteolytic cleavage following the release of extracellular proteases. The latter processes will not only take much longer periods of time, as compared to resistance induced by conventional antibiotics, but also have the potential to compromise cell wall integrity and are therefore detrimental to bacterial survival.

Recently, we have reported the identification of a cecropin-like AMP from the dengue virus-infected salivary glands of *Aedes aegypti* [10] for which the term Aedesin is coined. The chemically synthesized form of this peptide with a length of 36 amino acid residues was found to possess antibacterial activity against *E. coli*. In the present study, we have refined the analysis of Aedesin structural characteristics using nuclear magnetic resonance spectroscopy analysis and have furthermore determined its antimicrobial effects against a large panel of multidrug resistant clinical bacterial isolates and susceptible control reference strains.

## Materials and Methods

### Peptide synthesis

The identification of the cecropin-like peptide AAEL000598 peptide was recently described [10]. The peptide, with the following sequence ^26^ *GGLKKLGKKLEGAGKRVFKASEKALPVVVGIKAIGK^61^* and referred to as Aedesin in the present study, was chemically synthesized by Proteogenix (Schiltigheim, France) using FMOC (N-(9 fluorenyl)methoxycarbonyl) chemistry. The peptide is numbered starting from ^26^ *G* till *K^61^*, the first 25 residues not being included as they correspond to the leader sequence. In addition, a peptide of identical amino acid composition, but with a scrambled sequence (*VAKGLIKGVKAKGELPAKGVFKGLKESIGKRAVLKG*) and referred to as VG26-61, was synthesized and used as a negative control. The peptides were purified by reverse-phase preparative HPLC on a C18 column (20×250 mm; Shim-pack) using an appropriate 0-90% water/acetonitrile gradient in the presence of 0.05% trifluoroacetic acid. The purity of both peptides was checked by mass spectrometry and was more than 95% (data not shown). The molecular mass of both peptides was determined by matrix-assisted laser desorption ionization time-of-flight mass spectrometer (Axima-CFR Plus; Shimadzu). The concentration of the peptides was determined using an UV spectrometer.

### Nuclear magnetic resonance spectroscopy

The NMR sample was prepared by dissolving the Aedesin in a mixture of 50% PBS pH 7.4/50% TFE at a concentration of 784 µM in a 3 mm tube. TFE-d3 was purchased from Euriso-top. For the experiment in D_2_O the sample was lyophilized and dissolved in a mixture of 50% D_2_O/50% TFE. Spectra were acquired on 700 MHz Avance Bruker spectrometer equipped with triple-resonance (^1^H, ^15^N, ^13^C) z-gradient cryo-probe. Experiments were recorded using the Bruker TOPSPIN pulse sequence library (v.2.1). For all experiments, the recycling delay was 1.5 sec. 2D-Nuclear Overhauser effect spectroscopy (NOESY) experiment with excitation sculpting water suppression were acquired at 283K and 302K, with 48 scans and 2048 (t2) ×512 (t1) data size, and 10.2 ppm spectral width. The NOE mixing time was 200 msec. 2D- Total correlation spectroscopy (TOCSY) experiments with excitation sculpting water suppression was acquired at 283K with 32 scans and 2048 (t2) ×512 (t1) data size, and 10.2 ppm spectral width. The mixing time was 60 msec. 2D-^15^N-^1^H HSQC with binomial water suppression was acquired at 283K with 1024 scans and 1500 (t2) ×128 (t1) data size, and 10.2 ppm for the ^1^H and 40 ppm for the ^15^N spectral width. 2D-^13^C-^1^H HSQC was acquired with a D_2_O/TFE (50/50%) sample at 283K with 512 scans and 2048 (t2) ×182 (t1) data size, and 10.2 ppm for the ^1^H and 80 ppm for the ^13^C spectral width. All spectra are referenced to the internal reference DSS (4,4-dimethyl-4-silapentane-1-sulfonic acid) [11].

NMR data were processed using Topspin software and were analyzed using strip-plots. Side chain assignments were carried out using 2D-NOESY and 2D-TOCSY experiments with D_2_O/TFE samples. The side chain ^1^H resonances were assigned, with the exception of Hδ-Hε of Lys residues, the Hζ of Phe43 and the Hγ of Leu50. The NH of the first Gly residue remained unassigned. ^15^N assignments were derived from the 2D-^15^N-^1^H HSQC, however, due to NH superimposition, the ^15^N resonances of Glu36, Ala38, Val42, Phe43, Ser46, Val54 and Ile56 were not assigned. ^13^C assignments were derived from the 2D-^13^C-^1^H HSQC but the Cα of residues Gly26, Leu31, Lys34, Leu35, Lys40 and Ile56, and the Cβ of residues Lys29, Lys30, Lys33, Lys34, Lys40 and Lys44 could not be assigned.

### Structure calculation

Structure calculations were carried out by using the programs CYANA and CNS. From the NOESY at 283K, NOEs were classified from strong, medium and weak, corresponding to 2.8, 3.6 and 4.4 Å upper bound constraints, respectively. Structure calculations were performed with CYANA (v. 2.1) [12] using the 372 distance restraints from 2D- NOESY experiments. The NH, Hα, ^15^N, ^13^Cα and ^13^Cβ chemical shifts were converted into 52 Φ/Ψ dihedral angle constraints using TALOS+ (v. 1.2).

CYANA was used to calculate 100 structures, of which the 20 conformers with the lowest target function were refined by CNS (v. 1.2) [13] using 1000 steps of torsion angle dynamics at 250 K and 1000 steps of slow cooling to 100K, followed by 200 steps of Powell minimization. The final 20 conformers were selected with the lowest NOE and dihedral angle violations, and are the structures discussed herein and deposited (PDBs). The final 20 structures contained no NOE violations greater than 0.3 Å and no dihedral angle constraint violations greater than 2°. Structures were validated using PROCHECK [14]. The structure of Aedesin has been deposited at the Protein Data Bank (www.rcsb.org), under the entry assigned accession code: 2MMM.

### Circular Dichroism (CD) analysis

CD spectroscopy was used to investigate the secondary structure adopted by Aedesin in membrane-mimetic environments (1, 5 and 100 mM sodium dodecyl sulfate (SDS)). CD analysis was performed using a Chirascan Circular Dichroism Spectrophometer (Applied photophysics, Surrey, United Kingdom) with a polarized selected quartz cuve of 0,5 mm path length at 20°C. Wavelength from 180 to 260 were measured with a step of 0,5 nm and a bandwith of 2 nm. CD spectra were generated from an average of five scans of each sample. The peptide concentration was 45 µM in phosphate buffer (pH 7.4) containing 137 mM NaF for all experiments. Percentage of helicity was calculated using CONTIN Software (http://dichroweb.cryst.bbk.ac.uk).

### Bacterial strains

Five susceptible reference (*E. coli* ATCC 25922, *A. baumannii* ATCC 17978, *P. aeruginosa* ATCC 27853, *E. faecalis* ATCC 700802 and *S. aureus* ATCC 25923) and nineteen human clinical multidrug-resistant (MDR) or extensively drug-resistant (XDR) [15] strains commonly involved in human infections were used for MIC determination for Aedesin (Table S1). Fifteen and four clinical MDR/XDR isolates were collected at the Department of Bacteriology of the Montpellier University Hospital (DBUH) and Paris Salpêtrière University hospital respectively from 2012 to 2014. Among these bacteria, we have selected three *A. baumannii*, three *P. aeruginosa*, five *E. coli*, two *K. pneumonia*, three *S. aureus* and three *E. faecium* isolates. According to routine procedures, species identification was performed using matrix-assisted laser desorption ionization–time of flight (MALDI-TOF) mass spectrometry (MS) system methods (Bruker Biotyper) and the phenotypes of resistance to antibiotics were determined by using the disk (Bio-Rad, Marne-la-Coquette, France) diffusion method according to guidelines edited by the European Committee on Antimicrobial Susceptibility Testing (http://www.eucast.org). Zone diameter results were interpreted based on breakpoints established for each bacteria species by the Antibiogram Committee of the French Society of Microbiology (http://www.sfm-microbiologie.org). The definition of MDR, XDR and pandrug-resistant (PDR) came from international consensus Multidrug-resistant, extensively drug-resistant and pandrug-resistant bacteria: an international expert proposal for interim standard definitions for acquired resistance [15].

### Antibacterial activity

The antimicrobial activity of antibiotics, Aedesin and the scrambled control peptide VG26-61 against bacterial strains was determined by measuring the minimal inhibitory concentration (MIC) which represents the lowest concentration of drug or peptide that inhibits bacterial growth, using a broth microdilution method in 96-well plates (Microtest Tissue Culture plate, FALCON). In brief, pre-cultures were prepared by inoculation of 3 mL Mueller-Hinton (MH) browth and incubation at 37°C overnight under shaking. The pre-cultures were diluted to 1/100 in 3 mL MH and incubated for an additional 4 h at 37°C. The first column of the plate was a negative growth control, containing only 0.1 mL of MH. Columns 2 and 11 contained each 0.05 mL of peptide with a final concentration range of 0.0625 to 32 µg/mL, obtained by successive dilution of the peptide in the MH medium. The diluted peptides were prepared in the plate at concentrations 2 times higher than the desired final concentrations followed by the addition of the same volume of inoculum (total volume 0.1 mL/well). Inocula were prepared to obtain a final OD of 0.001 at a wavelength of 600 nm (Infinite F200 PRO, TECAN, Lyon, France), corresponding to 10^6^ CFU/mL. The last column of the plate was a positive growth control (without peptide), containing 0.05 mL of inoculum plus 0.05 mL of MH. The plates were incubated at 37°C for 22–24 h prior to the determination of the MIC, corresponding to the lowest concentration of drug or peptide necessary for preventing bacterial growth as visually observed (no growth viewed from the back of the plate against a dark background illuminated with reflected light) and confirmed by OD measurement at 600 nm in a plate reader (Infinite F200 PRO, TECAN).

Gentamicin and tobramycin were used for the susceptibility testing with *Escherichia coli* ATCC 25922 strain as an internal control. The breakpoints were determined using the European Committee on Antimicrobial Susceptibility Testing (EUCAST).

### Bactericidal activity

Non-treated bacteria were cultured to mid-log phase at 37°C in MH medium, spun for 10 minutes at 1000 g, resuspended in MH medium and diluted to an OD at 600 nm of 0.30 corresponding to 10^8^ CFU/ml. The bacteria were then grown for an additional 13 h in the absence or the presence of Aedesin followed by the determination of the OD at 600 nm.

### Analysis of the results

To evaluate the reproducibility of the assay, independent tests were performed using the susceptible referent strains (three tests) and the 19 clinical isolates (three tests). The reproducibility value was defined as the percentage of strains which gave the same MIC±1 log_2_ dilution at each test. The lecture of the MIC was performed by two independent operators.

### Transmission Electron Microscopy

Treated bacterial pellets were washed in phosphate buffered saline, fixed overnight in 2.5% glutaraldehyde (Electron Microscopy Sciences, Hatfield, US) and in 0.1 M sodium phosphate buffer at 4°C. Cells were post-fixed in 1% osmic acid (Electron Microscopy Sciences) for 1 hour at 4°C and with 0.5% tannic acid (Merck-Millipore, Darmstad, Germany) at 4°C for 30 min. Bacteria were dehydrated in a graded series of ethanol solutions (70/90/100%) for 30 min, embedded into resin and left to polymerize at ambient temperature for 1 h. Resins were sectioned by cutting an 80 nm film at 25°C using an ultramicrotome Ultracut Reichert (Leica Microsystemes SAS, Nanterre, France). Imaging was carried out using a Hitachi H1700 transmission electron microscope (Hitachi, Verrières-le-Buisson, France).

### Scanning Electron Microscopy

The morphological changes of bacterial cells, either untreated or incubated with Aedesin or the scrambled control peptide VG26-61, were determined by Scanning Electron Microscopy. Bacteria were spun at 300 g for 30 min after which the pellets were washed three times with phosphate buffered saline and deposited in 12 well plates. Samples were observed using a Hitachi S4000 electron microscope.

## Results

### NMR structure of GK 26-61

**Figure 1:**
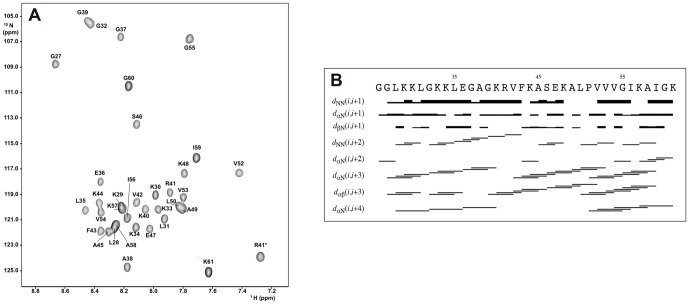
NOESY spectrum and NOE connectivities of Aedisine. (A) ^15^N-^1^H HSQC spectrum of Aedesin (G21- K61) at 50% TFE, pH 7.4 and 283K (mixing time, 200 ms). * indicates side chain NεH. (B) Schematic representation of NOE connectivities for Aedesin in 50% TFE. The intensity of the connectivity is reflected by the thickness of the bars.

Both NOESY and TOCSY spectra were collected for Aedesin at 283K, pH 7.4 in 50% TFE. The spin systems were identified based on the TOCSY spectrum with a mixing time of 60 ms and sequential assignments were obtained using the NOESY spectrum with a mixing time of 200 ms. Figure 1A shows the assignments of the ^15^N-^1^H cross peaks for GK 26-61. The ^15^N resonances of Glu36, Ala38, Val42, Phe43, Ser46, Val54 and Ile56 could not be unambiguously assigned and are represented according to standard amino-acid ^15^N chemical shifts. The (i, i+3) NOE connectivities denote an α-helical structure. Two stretches of dαN(i, i+4) NOE connectivities indicate the presence of regular α-helical conformation in the Leu^28^-Lys^40^ and Pro^51^-Lys^61^ regions. A summary for the sequential and medium range distance constraints for Aedesin in 50% TFE is shown in Figure 1B.

**Figure 2:**
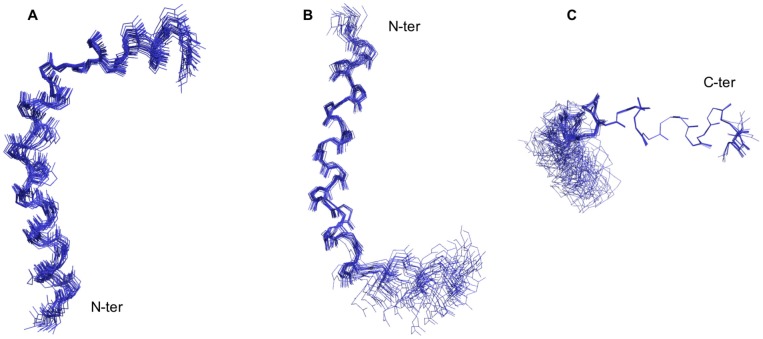
Calculated structures of Aedesin. (A) Superimposition of the 20 structures of Aedesin using backbone atoms. (B,C) the structures were aligned by two sections which are helix 1 from residues Lys^30^ to Lys^48^, and helix 2 from residues Val^52^ to Ile^59^, respectively.

**Figure 3:**
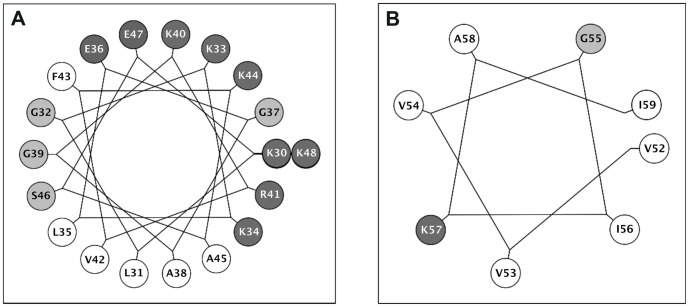
Helical wheel diagrams of Aedesin. (A) N-terminal helix region (helix 1) from Lys^30^ to Lys^48^ and (B) C-terminal helix (helix 2) from Val^52^ to Ile^59^. The hydrophobic or charged residues are indicated in black letters within the white circles and white letters within the dark grey circles, respectively. Other residues, including non-polar amino acids, are indicated in the light grey circles.

The solution structure of Aedesin was calculated using 372 NOE constraints derived from the NOESY spectrum at 283K. Dihedral angle constraints were obtained from NH, Hα, ^15^N, ^13^Cα and ^13^Cα chemical shifts data converted into 52 Φ/Ψ dihedral angle constraints using TALOS^+^. The analysis of the 20 overlapping structures of Aedesin (Figure 2) shows that the helical conformation is roughly continuous with a bent at residues 49-51, whereas those of the 20 final structures resulted in a Root-mean-square deviation (RMSD) of 0.846 Å for the backbone atoms and 1.597 Å for the heavy atoms. The structure of Aedesin is depicted as a helix-bent-helix structure with good RMSD statistics for the N-terminal helix (helix 1) and for the C-terminal helix (helix 2) taken separately. Structural statistics and the root mean square deviations for the 20 lowest energy structures of Aedesin are given in Table 1. The Ramachandran plot computed by PROCHECK shows that all the residues fall in the allowed conformational regions. Three amino acids, namely Ala-49, Leu-50 and Pro-51, are in the helical region of the Ramachandran plot. This also supports the NOE data obtained for these residues with the presence of dαN(i, i+3) and dαβ(i, i+3) NOE connectivities. The helical wheel diagram of Aedesin shows the amphipathic character of the first and second α-helices, as well as the opposite localization of their hydrophobic and positively charged residues, respectively (Figure 3). Whereas helix 1 has a prevalence of hydrophilic charged residues and a rather reduced hydrophobic side, the short second helix has a hydrophobic surface that consists of two Val and two Ile residues, indicating a stronger hydrophobic potential than the first helix.

**Table 1:** Summary of structural constraints and structure statistics.

**NOE constraints**	
Intraresidues (|i-j| = 0)	75
Sequential (|i-j| = 1)	142
Medium range (2≤|i-j|≤4)	155
Long range (|i-j|>4)	0
**Dihedral angles**	52
**Structural Statistics (20 Structures)**	
NOE violations, number >0.3 Å	0
Dihedral angle violations >2°	0
**RMSD for geometrical analysis**	
Bond lengths (Å)	0.0026+/−0.00013
Bond angles (degree)	0.4057+/−0.0077
Improper (degree)	0.3294+/−0.0151
**RMSD from experimental constraints**	
Distance (Å)	0.0293+/−0.0011
Dihedral angle (degree)	0.1659+/−0.0311
Mean total energy (kcal.mol-1)	83.82+/−5.60
**Atomic RMSD**	
Overall (26–61)	
Backbone	0.846
Heavy atoms	1.597
**Helix 1 (30–48)**	
Backbone	0.534
Heavy atoms	1.409
**Helix 2 (52–59)**	
Backbone	0.047
Heavy atoms	0.658

### Circular dichroism measurements

**Figure 4:**
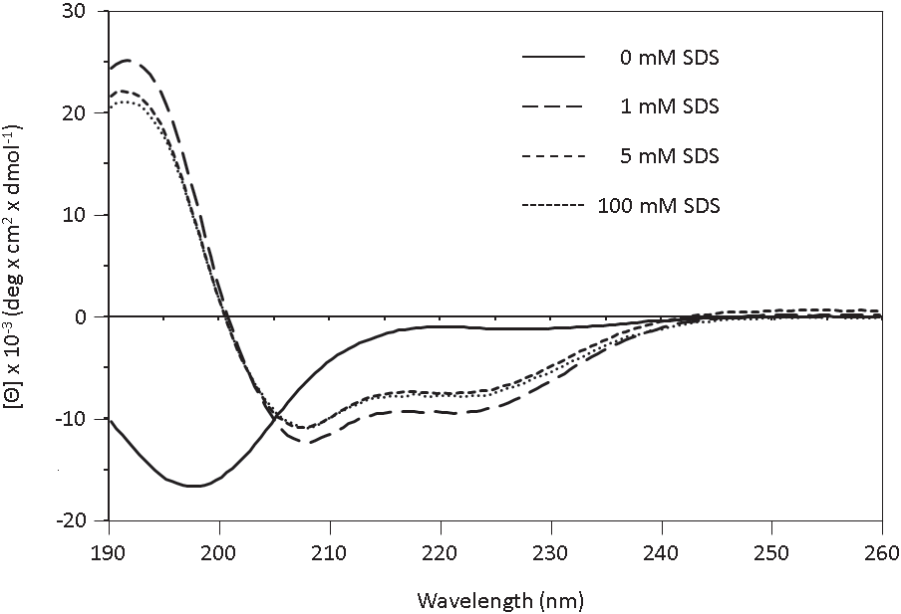
Circular dichroism of Aedesin in the presence of SDS micelles. CD spectra of the peptide were measured in phosphate buffer containing 137 mM NaF at SDS concentrations of 0, 1, 5 and 100 mM.

To investigate the secondary structure of Aedesin in a membrane-like environment, we analyzed the CD spectra of the peptide dissolved under increasing concentrations of SDS leading to the formation of micelles. As shown in Figure 4, the CD spectrum of Aedesin exhibited double minimum bands at 208 and 222 nm which indicate that Aedesin adopted a well-defined α-helical structure, already in the presence of 1 mM SDS, with a total helix content of 30% which remained stable also at concentrations of 5 and 100 mM SDS, respectively. In contrast, in the absence of SDS, the peptide was unable to form an α-helical structure.

### Antimicrobial activity of Aedesin

The antimicrobial activity of the Aedesin was determined on a comprehensive series of pathogenic, non-resistant, as well as MDR or XDR, Gram-positive and Gram-negative, bacterial isolates, commonly involved in human infections. The resistance phenotypes of each of the MDR or XDR strains, including *S. aureus*, *E. faecalis*, *E. faecium*, *E. coli*, *P. aeruginosa*, *A. baumannii* and *K. pneunomiae* to different classes of antibiotics is recapitulated in Table S1. In particular, the *E. coli* NMD1 and OXA48, as well as the *K. pneunomiae* KPC and VIM strains were selected because of the serious problems that they cause in the clinic, being resistant to the latest generation of antibiotics. Aedesin displayed strong anti-bacterial activity against all sixteen Gram-negative strains tested, independent of their antibiogram, as demonstrated by the low MIC values ranging between 1 and 4 (Table 2). In contrast, no antibacterial effects of the peptide were observed against different isolates of Gram-positive *S. aureus*, *E. faecalis* and *E. faecium* strains showing MIC values over 32. The scrambled control peptide VG26-61 was totally ineffective, irrespective of the bacterial strain.

**Table 2:** Antimicrobial activities of Aedesin against MDR bacterial strains.

Isolates	MIC (µg/mL) of Aedesin	MIC (µg/mL) of VG26-61
***E. coli***		
ATCC 25922	2 (1–2)	>32
EcESBL1	4 (2–4)	>32
Ec2	4 (2–4)	>32
EcESBL3	4 (2–4)	>32
EcNMD1	2	>32
EcOXA48	2	>32
***P. aeruginosa***		
ATCC 27853	4 (2–4)	>32
Pa1	1	>32
Pa2	2	>32
Pat3	1	>32
***A. baumannii***		
ATCC 17978	2	>32
Ab1	2	>32
Ab2	2	>32
Ab3	1	>32
***K. pneumoniae***		
KpKPC	2	>32
KpVIM	1	>32
***S. aureus***		
ATCC 25923	>32	>32
MRSA1	>32	>32
MRSA2	>32	>32
MRSA3	>32	>32
***Enterococcus***		
ATCC 700802	>32	>32
EfmGRE1	>32	>32
EfmGRE2	>32	>32
EfmGRE3	>32	>32

High salt concentrations are known to interfere with electrostatic contact between AMP and the negatively charged bacterial membrane, thereby potentially inhibiting their anti-microbial effects. To determine the activity of Aedesin in such an environment, the peptide was tested for salt resistance in the presence of either 150 mM NaCl, 1 mM MgCl_2_, 1 mM CaCl_2_, or a combination of these salts. Under these experimental conditions, Aedesin still showed a strong antimicrobial effect against all Gram-negative MDR strains, with MIC values between 1 and 2, indicating that its mode of action is maintained in a high salt environment (Table 3).

**Table 3:** Salt resistance of Aedesin.

Added salt	Concentration (mM)	*E. coli*		*P. aeruginosa*	
		MIC (µg/mL) of Aedesin	MIC (µg/mL) of VG26-61	MIC (µg/mL) of Aedesin	MIC (µg/mL) of VG26-61
**none**	-	1	>32	1–2	>32
**NaCl**	150	1	>32	1	>32
**CaCl_2_**	1	1	>32	1	>32
**MgCl_2_**	1	2	>32	2	>32
**NaCl +**	150	1	>32	1	>32
**CaCl_2_ +**	1				
**MgCl_2_**	1				

### Aedesin has bactericidal activity

The bactericidal activity of Aedesin was determined against two different MDR bacterial strains by measuring the viability following culture in the presence of either the Aedesin or the VG26-61 control peptide. Following a 13 h culture of *E. coli* and *P. Aeruginosa* in the presence of Aedesin at a concentration of 2 µg/mL, the OD_600_ diminished from 0.35±0.04 at the onset of the cultures to 0.17±0.01 and 0.11±0.01, respectively. In contrast, culture of the bacteria in the presence of the VG26-61 did not have any effect on their growth, with OD_600_ values of 1.2±0.01 and 1.5±0.01, respectively, at the end of the cultures.

### Effect of Aedesin treatment on the morphology of *E. coli*

**Figure 5:**
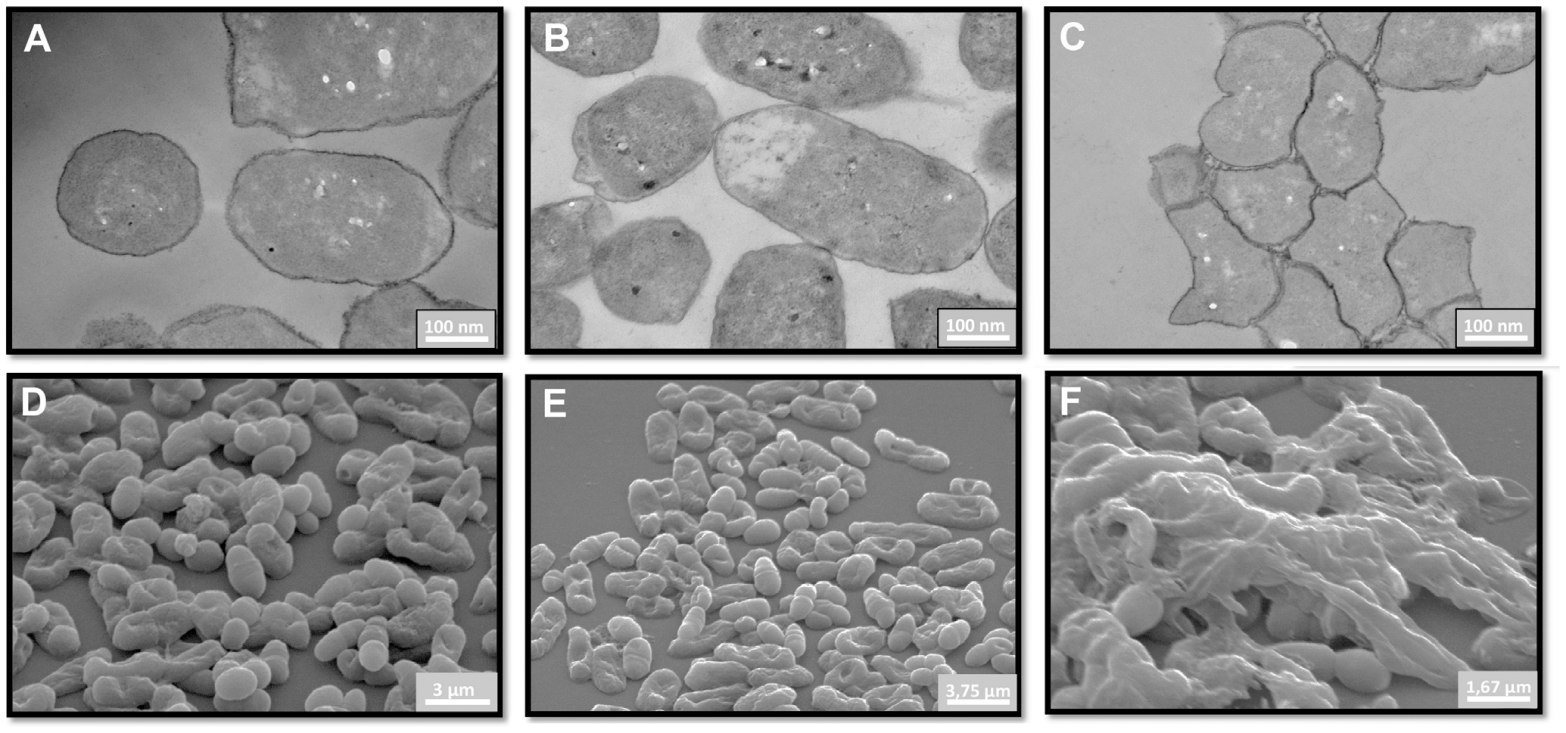
Electron microscopic analysis of Aedesin-treated bacteria. *E. coli* were either untreated (A,D) or incubated with VG26-61 (B,E) or Aedesin (C,F), respectively for 2 h at 37°C, prepared as indicated in Materials and Methods and analyzed by transmission (A–C) and scanning (D–F) electron microscopy.

*E. coli* treated with phosphate buffer only or with the scrambled control peptide VG26-61 had an intact outer membrane and displayed a regular cytoplasm, as shown by transmission electron microscopy analysis (Figure 5A,B). However, exposure of the bacteria to Aedesin resulted in strong aggregation and an important alteration of their cell membrane (Figure 5C). The strongly altered surface morphology of the bacteria treated with Aedesin was even more evident following analysis by scanning electron microscopy (Figure 5D–F).

## Discussion

Infections caused by MDR bacterial strains, resistant to even the latest class of antibiotics, have become a serious and worldwide problem. This is the consequence of a variety of microbial mechanisms, including production of enzymes that modify or destroy the active components of the antibiotic (by far the most prevalent mechanism), modification of the metabolic pathways that are antibiotic targets, as well as reduction of drug accumulation by rendering the bacterial cell wall impermeable for the antibiotic or by increasing active efflux of antibiotics across the cell surface [16]. Because of their particular mechanism of action, which is associated with a decreased tendency to induce bacterial resistance, AMP have gained considerable interest over the past decade as a possible alternative means to combat multidrug-resistance. In the present study, we have determined the antimicrobial capacity of one such AMP, denominated Aedesin, a cecropin-like peptide derived from the saliva of DENV-infected *Aedes aegypti* mosquitos [10]. In insects, cecropins form a large family of cationic α-helical peptides that are active mainly against Gram-negative bacteria [17], [18], [19], [20], [21]. Indeed, similar to CecropinA, Aedesin was found to be selective for Gram-negative bacteria and to efficiently kill a wide variety of MDR bacterial strains, including *P. aeruginosa*, *A. baumannii*, *K. pneumoniae* and *E. coli* with MIC values between 1 and 2 µg/mL.

The antimicrobial activity of certain AMPs, such as human β-defensins and the major human cationic host defense peptide LL-37, is strongly antagonized in conditions characterized by high ionic concentrations, which might preclude their therapeutic use in serum or other bodily fluids. For example, human β–defensins, as well as the major human cationic host defense peptide LL-37, are rapidly inactivated in the NaCl concentrations present in the airway surface liquid of cystic fibrosis patients [22], whereas interactions between cationic peptides and the outer surface component of Gram-negative bacteria are inhibited in the presence of high concentrations of bivalent ions [23]. However, the strong anti-bacterial activity of Aedesin was not affected by the presence of NaCl, MgCl_2_, CaCl_2,_ or a combination of these salts, at concentrations similar to those present in human bodily fluids [24], indicating that its killing mechanism is salt-resistant. Moreover, Aedesin is not toxic for human cells, at any of the concentrations used [10], further indicating that this AMP might have potential therapeutic use in a physiological environment. It is to be stressed however that peptides, and in particular AMP, have poor *in vivo* stability, in particular when composed of L-amino acids, and are readily disintegrated by proteolytic enzymes in bodily fluids or recognized and processed by tissue-resident antigen-presenting cells which limits their systemic therapeutic use. Moreover, renal clearance limits the *in vivo* half-life of peptides in the circulation to only a few hours [25]. These considerations notwithstanding, several approaches that impede proteolysis in serum conditions, while retaining the bactericidal activity of the AMP, have been reported, such as substitution of L- by D-amino acids, cyclization of the peptides, use of fluorinated amino acids, beta peptides or conjugation of fatty acids [26]. Another strategy is the substitution of certain residues by unusual amino acids. For example, the replacement of Arg residues within the Oncocin-1 peptide by Orn [27] or Arg substitution within cationic amphiphilic or cationic polypeptides by Aib and Agp residues [28] were shown to confer protection to degradation and improve serum stability. A detailed structure-function analysis of Aedesin therefore needs to be carried out to determine, and possibly ameliorate, the pharmacokinetics and bioavailability of this peptide for systemic use.

The results from CD analysis of the peptide in the presence of increasing concentrations of SDS showed that Aedesin readily adopts a helical structure in a hydrophic environment. This finding was confirmed and extended by the results from NMR analysis demonstrating that Aedesin consists of two regular amphipatic α-helices in the Lys^30^-Lys^48^ and Val^52^-Ile^59^ regions, respectively, at the N- and C-terminal part of the peptide. The N-terminal region contains a large stretch of positively charged residues including six Lys residues. In contrast, the C-terminal helix is clearly hydrophobic with two Ile and three Val residues, separated by a single charged Lys. Although the presence of a helix-hinge-helix is a common feature found in many cecropin family members, this property does not guarantee its antibacterial activity. For example, cecropin B1, although sharing a similar conformation with Aedesin, has poor anti-microbial activity [29], underscoring the correct composition and distribution of key amino acid residues in Aedesin that are critical for this function.

Our results from scanning electron microscopic analysis show that Aedesin strongly alters the bacterial morphology, indicating that it exerts its lytic function by disrupting the bacterial outer membrane of Gram-negative bacteria. Similar results have been reported for Cecropins B, D [19], as well as Cecropin A. Indeed, using lipid vesicles with varying phospholipid composition, mimicking mammalian or microbial membranes, Cecropin A was found to preferentially permeate microbial or fungal membranes characterized by the presence of negatively charged phospholipids, rather than zwitterionic phospholipid-containing mammalian membranes [29]. In this respect, it is of note that Aedesin also kills the parasite *Leishmania donovani* [10], which is in agreement with previously published reports that demonstrate the presence of high amounts of lipophosphoglycan molecules in the membrane of this promastigote [30], [31], thus forming a protective anionic barrier shielding that is sensitive to cationic molecules or ionizable phospholipid groups that cause destabilization of the membrane [32].

Like other cecropins, Aedesin is ineffective against various MDR *S. aureus* strains, thus corroborating the notion that the cytoplasmic membranes of Gram-positive bacteria are inherently more resistant to these cationic peptides, as compared to Gram-negative microorganisms [19]. Indeed, the interaction between the peptide and the bacterial membrane is determined by the lipid composition of the membrane, its surface charge density and by the presence of an electrochemical potential across the membrane, underscoring the difference between the membrane components, resulting in their differential sensitivity to membrane permeabilization by cationic peptides, between both groups of bacteria.

In conclusion, the results of this study show that the killing of MDR bacterial strains by Aedesin is independent from most mechanisms of bacterial resistance. Although it is unlikely, in its present form, to be used to systemically treat MDR Gram-negative bacterial infections, the topical use of this cationic AMP could be envisaged. For example, polymyxin B and E, while toxic at clinical doses for systemic use as anti-bacterial drugs, have been successfully implemented in the treatment of cutaneous infections caused by *P. aeruginosa* and *A. baumannii* [25], both strains that are highly susceptible to the bactericidal effects of Aedesin, as shown in the present study. Certain AMP have also been formulated in artificial tear solutions, lens preservation fluid and generic wound creams [33]. Of great interest is the application of cationic peptides against biofilm-forming bacterial infections. In particular, their application as nanofilms or other coating materials for surgical devices, including catheters and medical implants are currently under study [34]. Substitution experiments to determine the essential amino acid residues involved in the lytic function of this peptide, while trying to preserve or ameliorate its stability are currently underway.

## Supporting Information

Table S1 Overview of bacterial isolates and antimicrobial susceptibility.
(TIFF)
